# (1*E*,4*E*)-1,5-Bis(2,4,6-trimeth­oxy­phen­yl)penta-1,4-dien-3-one

**DOI:** 10.1107/S1600536810049299

**Published:** 2010-12-04

**Authors:** Pumsak Ruanwas, Suchada Chantrapromma, Hoong-Kun Fun

**Affiliations:** a Crystal Materials Research Unit, Department of Chemistry, Faculty of Science, Prince of Songkla University, Hat-Yai, Songkhla 90112, Thailand; b Department of Chemistry and Center of Excellence for Innovation in Chemistry, Faculty of Science, Prince of Songkla University, Hat-Yai, Songkhla 90112, Thailand; c X-ray Crystallography Unit, School of Physics, Universiti Sains Malaysia, 11800 USM, Penang, Malaysia

## Abstract

There are two crystallographically independent mol­ecules in the asymmetric unit of the title bis­chalcone derivative, C_23_H_26_O_7_. The mol­ecules are unsymmetrical and almost planar: the dihedral angle between two benzene rings is 1.04 (7)° in one mol­ecule and 2.31 (7)° in the other. The central penta-1,4-dien-3-one fragment makes dihedral angles of 7.61 (7) and 6.82 (7)° with the two adjacent benzene rings in one mol­ecule, while the corresponding values are 7.85 (7) and 9.42 (6)° in the other. In both mol­ecules, the three meth­oxy groups of the two 2,4,6-trimeth­oxy­phenyl units are coplanar with the attached benzene rings [C—O—C—C- torsion angles of −1.5 (2), −7.2 (2) and 4.1 (2)° in one mol­ecule and −0.7 (2), −5.5 (2) and −0.6 (2)° in the other]. The mol­ecular conformations are stabilized by weak intra­molecular C—H⋯O inter­actions generating two *S*(6) ring motifs. In the crystal, mol­ecules are linked by weak inter­molecular C—H⋯O inter­actions into zigzag chains parallel to the *c* axis. The crystal structure is further stabilized by C—H⋯π inter­actions and π–π inter­actions with centroid–centroid distances of 3.6433 (8) Å.

## Related literature

For bond-length data, see: Allen *et al.* (1987 ▶). For hydrogen-bond motifs, see: Bernstein *et al.* (1995 ▶). For related structures, see: Fun *et al.* (2010 ▶); Harrison *et al.* (2006 ▶). For background to and applications of bis­chalcones, see: Gomes *et al.* (2009 ▶); Lee *et al.* (2009 ▶); Quincoces *et al.* (2008 ▶); Uchida *et al.* (1998 ▶). For the stability of the temperature controller used in the data collection, see: Cosier & Glazer (1986 ▶).
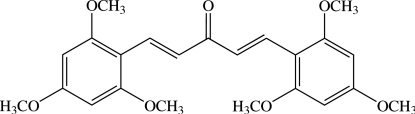


## Experimental

### 

#### Crystal data

C_23_H_26_O_7_
*M* *_r_* = 414.44
Monoclinic, 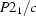

*a* = 15.7417 (2) Å
*b* = 15.1192 (2) Å
*c* = 19.4803 (3) Å
β = 117.827 (1)°
*V* = 4100.21 (11) Å^3^
*Z* = 8
Mo *K*α radiation
μ = 0.10 mm^−1^
*T* = 100 K
0.39 × 0.32 × 0.17 mm

#### Data collection

Bruker APEXII DUO CCD area-detector diffractometer
Absorption correction: multi-scan (*SADABS*; Bruker, 2005 ▶) *T*_min_ = 0.963, *T*_max_ = 0.983
50384 measured reflections
10908 independent reflections
7903 reflections with *I* > 2σ(*I*)
*R*_int_ = 0.032

#### Refinement

*R*[*F* ^2^ > 2σ(*F* ^2^)] = 0.052
*wR*(*F* ^2^) = 0.139
*S* = 1.04
10908 reflections
553 parameters
H-atom parameters constrained
Δρ_max_ = 0.36 e Å^−3^
Δρ_min_ = −0.24 e Å^−3^

### 

Data collection: *APEX2* (Bruker, 2005 ▶); cell refinement: *SAINT* (Bruker, 2005 ▶); data reduction: *SAINT*; program(s) used to solve structure: *SHELXTL* (Sheldrick, 2008 ▶); program(s) used to refine structure: *SHELXTL*; molecular graphics: *SHELXTL*; software used to prepare material for publication: *SHELXTL* and *PLATON* (Spek, 2009 ▶).

## Supplementary Material

Crystal structure: contains datablocks global, I. DOI: 10.1107/S1600536810049299/rz2523sup1.cif

Structure factors: contains datablocks I. DOI: 10.1107/S1600536810049299/rz2523Isup2.hkl

Additional supplementary materials:  crystallographic information; 3D view; checkCIF report

## Figures and Tables

**Table 1:** Hydrogen-bond geometry (Å, °) *Cg*1, *Cg*2 and *Cg*3 are the centroids of the C1*A*–C6*A*, C12*A*–C17*A* and C12*B*–C17*B* rings, respectively

*D*—H⋯*A*	*D*—H	H⋯*A*	*D*⋯*A*	*D*—H⋯*A*
C7*A*—H7*AA*⋯O1*A*	0.93	2.42	2.787 (2)	104
C7*A*—H7*AA*⋯O2*A*	0.93	2.29	2.704 (2)	106
C8*A*—H8*AA*⋯O4*A*	0.93	2.18	2.7844 (17)	121
C7*B*—H7*BA*⋯O1*B*	0.93	2.49	2.8351 (19)	102
C7*B*—H7*BA*⋯O2*B*	0.93	2.33	2.704 (2)	104
C8*B*—H8*BA*⋯O4*B*	0.93	2.16	2.7643 (18)	121
C10*A*—H10*A*⋯O5*A*	0.93	2.26	2.852 (2)	121
C10*B*—H10*B*⋯O5*B*	0.93	2.26	2.855 (2)	121
C11*A*—H11*A*⋯O7*A*	0.93	2.23	2.6599 (17)	108
C11*B*—H11*B*⋯O7*B*	0.93	2.23	2.6700 (17)	108
C20*A*—H20*C*⋯O1*B*	0.96	2.47	3.339 (2)	151
C20*B*—H20*E*⋯O1*A*^i^	0.96	2.37	3.0238 (19)	125
C23*A*—H23*C*⋯O1*A*^ii^	0.96	2.39	3.319 (2)	162
C23*B*—H23*D*⋯O1*B*^ii^	0.96	2.41	3.262 (2)	148
C18*A*—H18*C*⋯*Cg*1^iii^	0.96	2.65	3.4503 (17)	141
C21*A*—H21*C*⋯*Cg*2^iv^	0.96	2.94	3.5813 (18)	126
C21*B*—H21*E*⋯*Cg*3^v^	0.96	2.72	3.5809 (16)	150

Symmetry codes: (i) 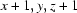; (ii) 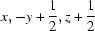; (iii) 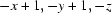; (iv) 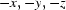; (v) 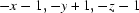.

**Fig. 1.**
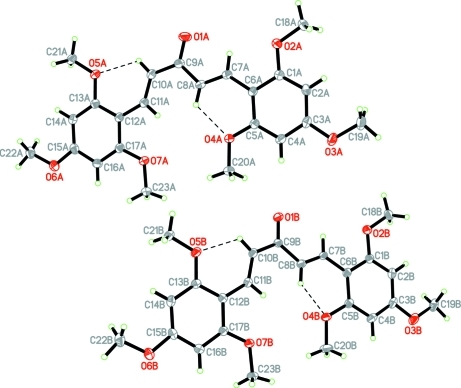
The molecular structure of the title compound, showing 50% probability displacement ellipsoids and the atom-numbering scheme. Intramolecular C—H···O interactions are shown as dashed lines.

**Fig. 2.**
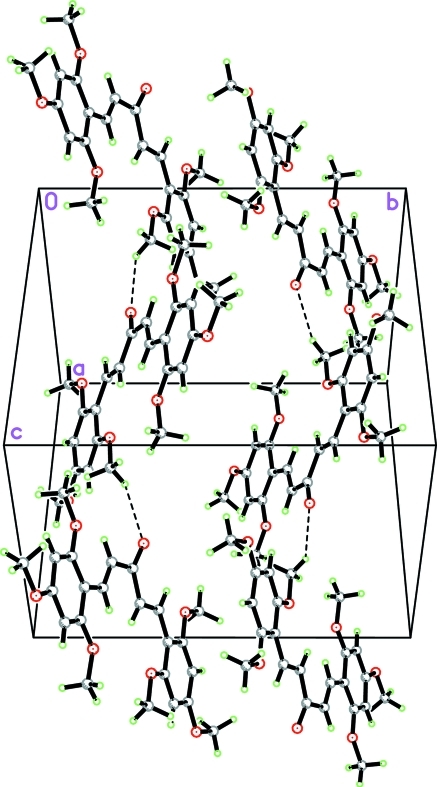
The crystal packing of the title compound showing the zigzag chains along the c axis. Hydrogen bonds are shown as dashed lines.

## supplementary crystallographic information

### Comment

Bischalcone is an important class of compounds due to their variety of properties such as non-linear optical (Uchida *et al.*, 1998) and fluorescence properties (Gomes *et al.*, 2009) and activities involving anti-inflammatory, antioxidant and anti-tyrosinase activities (Lee *et al.*, 2009) and cytotoxic activities (Quincoces *et al.*, 2008). We have previously reported the crystal structure of (1*E*,4*E*)-1,5-bis(2,4,5-trimethoxyphenyl)penta-1,4-dien-3-one (I) (Fun *et al.*, 2010). The title bischalcone (II) was synthesized in order to study the effect of the positions of the trimethoxy substituents to its fluorescence property and anti-tyrosinase activity. Our anti-tyrosinase activity testing showed that the title bischacone possesses anti-tyrosinase activity. We report herein the crystal structure of (II).

There are two crystallograpich independent molecules (*A* and *B*) in the asymmetric unit of (II) (Fig. 1) with the same conformation but with slight differences in bond angles. The molecular structure of (II) is unsymmetrical and almost planar. The dihedral angle between the C1–C6 and C12–C17 benzene rings is 1.04 (7)° in molecule *A* whereas it is 2.31 (7)° in molecule *B*. The central penta-1,4-dien-3-one unit (C7–C11/O1) is planar with *r.m.s.* of 0.0124 (1) and 0.0433 (1) Å for molecule *A* and *B*, respectively. This unit makes a dihedral angles of 7.61 (7) and 6.82 (7)° with the two adjacent C1–C6 and C12–C17 benzene rings, respectively, in molecule *A* whereas the corresponding values are 7.85 (7) and 9.42 (6)° in molecule *B*. The three methoxy groups on the C1–C6 benzene ring are planarly attached, with the C18–O2–C1–C2, C19–O3–C3–C2 and C20–O4–C5–C4 torsion angles of -1.5 (2), -7.2 (2) and 4.1 (2)° in molecule *A* and -0.7 (2), -5.5 (2) and -0.6 (2)° in molecule *B*. The same orientation is observed for the three methoxy groups on the C12–C17 benzene ring as indicated by the torsion angles C21–O5–C13–C14, C22–O6–C15–C14 and C23–O7–C17–C16 of -0.1 (2), -0.7 (2) and 0.8 (2)°, respectively, for molecule *A* and the corresponding values of -0.9 (2), 1.6 (2) and -4.9 (2)° for molecule *B*. In each molecule, intramolecular C8A—H8AA···O4A and C10A—H10A···O5A [in molecule *A*]; C8B—H8BA···O4B and C10B—H10B···O5B [in molecule *B*] weak interactions (Table 1, Fig. 1) generate S(6) ring motifs (Bernstein *et al.*, 1995). The bond distances are in normal ranges (Allen *et al.*, 1987) and are comparable with those of related structures (Fun *et al.*, 2010; Harrison *et al.*, 2006). However there are less C—H···O weak interactions but more C—H···π interactions in (II) than in (I).

In the crystal packing (Fig. 2), the molecules are linked by intermolecular C—H···O weak interactions (Table 1) into zigzag chains along the *c* axis. The crystal is stabilized by intermolecular C—H···O weak interactions and C—H···π interactions (Table 1). π–π interactions are observed with *Cg*_1_···*Cg*_3_^i^ = 3.6433 (8) Å (symmetry code: (i) *x*, 1/2 - *y*, -1/2 + *z*; *Cg*_1_ and *Cg*_3_ are the centroids of the C1A–C6A and C12B–C17B rings, respectively).

### Experimental

The title compound was synthesized by dissolving the 2,4,6-trimethoxybenzaldehyde (0.5 g, 2.55 mmol) in acetone (50 ml). A NaOH 50% aqueous solution (2 ml) was then added and, after stirring at room temperature for 1h, the resulting yellow solid was collected by filtration, washed with distilled water and dried. Pale yellow block-shaped single crystals of the title compound suitable for *x*-ray structure determination were recrystalized from ethanol by the slow evaporation of the solvent at room temperature after a week. M. p. 494–495 K.

### Refinement

All H atoms were positioned geometrically and allowed to ride on their parent atoms, with d(C—H) = 0.93 Å for aromatic and 0.96 Å for CH_3_ atoms. The *U*_iso_ values were constrained to be 1.5*U*_eq_ of the carrier atom for methyl H atoms and 1.2*U*_eq_ for the remaining H atoms. A rotating group model was used for the methyl groups. The highest residual electron density peak is located at 0.70 Å from C12B and the deepest hole is located at 1.24 Å from C13B.

### Crystal data

**Table:**

C_23_H_26_O_7_	*F*(000) = 1760
*M**_r_* = 414.44	*D*_x_ = 1.343 Mg m^−^^3^
Monoclinic, *P*2_1_/*c*	Melting point = 494–495 K
Hall symbol: -P 2ybc	Mo *K*α radiation, λ = 0.71073 Å
*a* = 15.7417 (2) Å	Cell parameters from 10908 reflections
*b* = 15.1192 (2) Å	θ = 2.1–29.0°
*c* = 19.4803 (3) Å	µ = 0.10 mm^−^^1^
β = 117.827 (1)°	*T* = 100 K
*V* = 4100.21 (11) Å^3^	Block, pale yellow
*Z* = 8	0.39 × 0.32 × 0.17 mm

### Data collection

**Table:**

Bruker APEXII DUO CCD area-detector diffractometer	10908 independent reflections
Radiation source: sealed tube	7903 reflections with *I* > 2σ(*I*)
graphite	*R*_int_ = 0.032
φ and ω scans	θ_max_ = 29.0°, θ_min_ = 2.1°
Absorption correction: multi-scan (*SADABS*; Bruker, 2005)	*h* = −20→21
*T*_min_ = 0.963, *T*_max_ = 0.983	*k* = −20→18
50384 measured reflections	*l* = −26→26

### Refinement

**Table:**

Refinement on *F*^2^	Primary atom site location: structure-invariant direct methods
Least-squares matrix: full	Secondary atom site location: difference Fourier map
*R*[*F*^2^ > 2σ(*F*^2^)] = 0.052	Hydrogen site location: inferred from neighbouring sites
*wR*(*F*^2^) = 0.139	H-atom parameters constrained
*S* = 1.04	*w* = 1/[σ^2^(*F*_o_^2^) + (0.0631*P*)^2^ + 1.5299*P*] where *P* = (*F*_o_^2^ + 2*F*_c_^2^)/3
10908 reflections	(Δ/σ)_max_ = 0.001
553 parameters	Δρ_max_ = 0.36 e Å^−^^3^
0 restraints	Δρ_min_ = −0.24 e Å^−^^3^

### Special details

**Table:**

Experimental. The crystal was placed in the cold stream of an Oxford Cryosystems Cobra open-flow nitrogen cryostat (Cosier & Glazer, 1986) operating at 100.0 (1) K.
Geometry. All e.s.d.'s (except the e.s.d. in the dihedral angle between two l.s. planes) are estimated using the full covariance matrix. The cell e.s.d.'s are taken into account individually in the estimation of e.s.d.'s in distances, angles and torsion angles; correlations between e.s.d.'s in cell parameters are only used when they are defined by crystal symmetry. An approximate (isotropic) treatment of cell e.s.d.'s is used for estimating e.s.d.'s involving l.s. planes.
Refinement. Refinement of *F*^2^ against ALL reflections. The weighted *R*-factor *wR* and goodness of fit *S* are based on *F*^2^, conventional *R*-factors *R* are based on *F*, with *F* set to zero for negative *F*^2^. The threshold expression of *F*^2^ > σ(*F*^2^) is used only for calculating *R*-factors(gt) *etc*. and is not relevant to the choice of reflections for refinement. *R*-factors based on *F*^2^ are statistically about twice as large as those based on *F*, and *R*- factors based on ALL data will be even larger.

### Fractional atomic coordinates and isotropic or equivalent isotropic displacement parameters (Å^2^)

**Table:**

	*x*	*y*	*z*	*U*_iso_*/*U*_eq_	
O1A	0.17578 (9)	0.23957 (10)	−0.12657 (6)	0.0395 (3)	
O2A	0.45629 (7)	0.37966 (7)	−0.07200 (6)	0.0219 (2)	
O3A	0.72990 (7)	0.41499 (7)	0.18177 (6)	0.0257 (2)	
O4A	0.43772 (7)	0.29181 (7)	0.15211 (5)	0.0209 (2)	
O5A	−0.05667 (7)	0.12267 (7)	−0.05942 (6)	0.0218 (2)	
O6A	−0.07167 (8)	0.03212 (8)	0.17332 (6)	0.0287 (3)	
O7A	0.20935 (7)	0.16050 (7)	0.18551 (6)	0.0233 (2)	
C1A	0.50181 (11)	0.37297 (9)	0.00690 (8)	0.0176 (3)	
C2A	0.59599 (11)	0.40105 (9)	0.05208 (9)	0.0196 (3)	
H2AA	0.6305	0.4261	0.0292	0.023*	
C3A	0.63756 (10)	0.39085 (10)	0.13221 (9)	0.0199 (3)	
C4A	0.58625 (10)	0.35517 (10)	0.16765 (8)	0.0200 (3)	
H4AA	0.6148	0.3497	0.2214	0.024*	
C5A	0.49247 (10)	0.32789 (9)	0.12218 (8)	0.0180 (3)	
C6A	0.44650 (10)	0.33541 (9)	0.03973 (8)	0.0169 (3)	
C7A	0.35023 (10)	0.30407 (9)	−0.01164 (8)	0.0180 (3)	
H7AA	0.3314	0.3105	−0.0643	0.022*	
C8A	0.28416 (10)	0.26743 (10)	0.00458 (8)	0.0191 (3)	
H8AA	0.2963	0.2625	0.0559	0.023*	
C9A	0.19240 (11)	0.23482 (10)	−0.05818 (8)	0.0209 (3)	
C10A	0.12089 (11)	0.19400 (10)	−0.04001 (8)	0.0204 (3)	
H10A	0.0631	0.1753	−0.0809	0.024*	
C11A	0.13474 (10)	0.18232 (9)	0.03281 (8)	0.0175 (3)	
H11A	0.1931	0.2040	0.0708	0.021*	
C12A	0.07555 (10)	0.14215 (9)	0.06315 (8)	0.0177 (3)	
C13A	−0.01944 (10)	0.11210 (9)	0.01869 (8)	0.0183 (3)	
C14A	−0.07145 (11)	0.07451 (10)	0.05295 (8)	0.0202 (3)	
H14A	−0.1340	0.0547	0.0226	0.024*	
C15A	−0.02790 (11)	0.06729 (10)	0.13332 (9)	0.0221 (3)	
C16A	0.06571 (11)	0.09625 (10)	0.17972 (9)	0.0224 (3)	
H16A	0.0937	0.0912	0.2334	0.027*	
C17A	0.11655 (10)	0.13254 (10)	0.14487 (8)	0.0193 (3)	
C18A	0.50745 (11)	0.41912 (10)	−0.10866 (9)	0.0235 (3)	
H18A	0.4664	0.4221	−0.1637	0.035*	
H18B	0.5630	0.3840	−0.0983	0.035*	
H18C	0.5273	0.4777	−0.0886	0.035*	
C19A	0.78857 (12)	0.44273 (12)	0.14764 (10)	0.0325 (4)	
H19A	0.8528	0.4535	0.1878	0.049*	
H19B	0.7627	0.4961	0.1185	0.049*	
H19C	0.7896	0.3973	0.1136	0.049*	
C20A	0.47734 (12)	0.28718 (12)	0.23484 (8)	0.0264 (3)	
H20A	0.4308	0.2620	0.2480	0.040*	
H20B	0.4938	0.3456	0.2564	0.040*	
H20C	0.5339	0.2509	0.2556	0.040*	
C21A	−0.15303 (11)	0.09395 (10)	−0.10841 (9)	0.0229 (3)	
H21A	−0.1695	0.1054	−0.1616	0.034*	
H21B	−0.1962	0.1255	−0.0951	0.034*	
H21C	−0.1580	0.0317	−0.1013	0.034*	
C22A	−0.16834 (11)	0.00175 (11)	0.13032 (10)	0.0271 (3)	
H22A	−0.1905	−0.0197	0.1655	0.041*	
H22B	−0.1710	−0.0451	0.0962	0.041*	
H22C	−0.2085	0.0497	0.1004	0.041*	
C23A	0.25648 (12)	0.15076 (12)	0.26804 (8)	0.0272 (4)	
H23A	0.3190	0.1774	0.2894	0.041*	
H23B	0.2629	0.0890	0.2811	0.041*	
H23C	0.2192	0.1793	0.2890	0.041*	
O1B	0.68800 (7)	0.20303 (8)	0.36386 (6)	0.0241 (2)	
O2B	0.99141 (7)	0.09844 (7)	0.41753 (6)	0.0225 (2)	
O3B	1.26319 (8)	0.06825 (8)	0.67341 (6)	0.0287 (3)	
O4B	0.96545 (7)	0.18131 (7)	0.63905 (6)	0.0223 (2)	
O5B	0.48221 (7)	0.36335 (7)	0.43616 (6)	0.0209 (2)	
O6B	0.47137 (8)	0.44271 (8)	0.67297 (6)	0.0260 (2)	
O7B	0.75115 (7)	0.31918 (7)	0.67890 (6)	0.0211 (2)	
C1B	1.03565 (10)	0.10545 (10)	0.49647 (8)	0.0185 (3)	
C2B	1.13074 (10)	0.07922 (10)	0.54267 (9)	0.0206 (3)	
H2BA	1.1665	0.0549	0.5205	0.025*	
C3B	1.17063 (10)	0.09028 (10)	0.62243 (9)	0.0213 (3)	
C4B	1.11777 (11)	0.12564 (10)	0.65642 (8)	0.0220 (3)	
H4BA	1.1457	0.1329	0.7100	0.026*	
C5B	1.02313 (10)	0.14997 (9)	0.60976 (8)	0.0184 (3)	
C6B	0.97838 (10)	0.14116 (9)	0.52777 (8)	0.0172 (3)	
C7B	0.87935 (10)	0.16500 (9)	0.47616 (8)	0.0169 (3)	
H7BA	0.8555	0.1456	0.4252	0.020*	
C8B	0.81720 (10)	0.21120 (9)	0.49162 (8)	0.0174 (3)	
H8BA	0.8372	0.2320	0.5418	0.021*	
C9B	0.71850 (10)	0.22977 (9)	0.43148 (8)	0.0164 (3)	
C10B	0.65299 (10)	0.28005 (9)	0.45157 (8)	0.0174 (3)	
H10B	0.5947	0.2990	0.4115	0.021*	
C11B	0.67345 (10)	0.29988 (9)	0.52518 (8)	0.0163 (3)	
H11B	0.7353	0.2846	0.5620	0.020*	
C12B	0.61648 (10)	0.34076 (9)	0.55742 (8)	0.0162 (3)	
C13B	0.52085 (10)	0.37092 (9)	0.51463 (8)	0.0169 (3)	
C14B	0.46987 (10)	0.40590 (9)	0.55087 (8)	0.0190 (3)	
H14B	0.4072	0.4259	0.5217	0.023*	
C15B	0.51478 (11)	0.41016 (9)	0.63141 (9)	0.0195 (3)	
C16B	0.60879 (11)	0.38162 (10)	0.67653 (8)	0.0194 (3)	
H16B	0.6376	0.3849	0.7304	0.023*	
C17B	0.65853 (10)	0.34824 (9)	0.63965 (8)	0.0171 (3)	
C18B	1.04507 (11)	0.06389 (11)	0.38133 (9)	0.0248 (3)	
H18D	1.0042	0.0593	0.3264	0.037*	
H18E	1.0694	0.0064	0.4021	0.037*	
H18F	1.0978	0.1027	0.3911	0.037*	
C19B	1.31903 (11)	0.02436 (11)	0.64289 (10)	0.0280 (4)	
H19D	1.3822	0.0125	0.6843	0.042*	
H19E	1.3238	0.0615	0.6049	0.042*	
H19F	1.2885	−0.0303	0.6191	0.042*	
C20B	1.00518 (12)	0.18825 (12)	0.72165 (8)	0.0293 (4)	
H20D	0.9576	0.2116	0.7344	0.044*	
H20E	1.0597	0.2271	0.7413	0.044*	
H20F	1.0249	0.1308	0.7446	0.044*	
C21B	0.38459 (10)	0.39154 (10)	0.38923 (8)	0.0226 (3)	
H21D	0.3672	0.3838	0.3354	0.034*	
H21E	0.3787	0.4528	0.3992	0.034*	
H21G	0.3427	0.3568	0.4018	0.034*	
C22B	0.37374 (12)	0.47159 (12)	0.63068 (10)	0.0315 (4)	
H22D	0.3517	0.4916	0.6664	0.047*	
H22G	0.3344	0.4233	0.6006	0.047*	
H22E	0.3697	0.5192	0.5967	0.047*	
C23B	0.80039 (12)	0.33050 (12)	0.76164 (8)	0.0273 (4)	
H23G	0.8652	0.3097	0.7818	0.041*	
H23D	0.7680	0.2974	0.7845	0.041*	
H23E	0.8009	0.3920	0.7740	0.041*	

### Atomic displacement parameters (Å^2^)

**Table:**

	*U*^11^	*U*^22^	*U*^33^	*U*^12^	*U*^13^	*U*^23^
O1A	0.0300 (7)	0.0677 (9)	0.0156 (5)	−0.0216 (6)	0.0063 (5)	0.0002 (6)
O2A	0.0209 (6)	0.0262 (6)	0.0191 (5)	−0.0032 (4)	0.0099 (4)	0.0011 (4)
O3A	0.0156 (5)	0.0298 (6)	0.0281 (6)	−0.0049 (4)	0.0072 (5)	−0.0042 (5)
O4A	0.0176 (5)	0.0289 (6)	0.0148 (5)	−0.0008 (4)	0.0064 (4)	0.0027 (4)
O5A	0.0171 (5)	0.0276 (6)	0.0170 (5)	−0.0012 (4)	0.0049 (4)	−0.0006 (4)
O6A	0.0247 (6)	0.0382 (7)	0.0270 (6)	−0.0021 (5)	0.0151 (5)	0.0055 (5)
O7A	0.0178 (5)	0.0333 (6)	0.0154 (5)	−0.0011 (4)	0.0049 (4)	0.0006 (4)
C1A	0.0195 (7)	0.0141 (7)	0.0196 (7)	0.0018 (5)	0.0094 (6)	−0.0001 (5)
C2A	0.0194 (7)	0.0167 (7)	0.0258 (7)	0.0003 (6)	0.0133 (6)	−0.0008 (6)
C3A	0.0143 (7)	0.0175 (7)	0.0252 (7)	0.0003 (6)	0.0069 (6)	−0.0035 (6)
C4A	0.0179 (7)	0.0214 (7)	0.0174 (7)	0.0017 (6)	0.0055 (6)	−0.0003 (5)
C5A	0.0173 (7)	0.0162 (7)	0.0197 (7)	0.0024 (5)	0.0080 (6)	0.0006 (5)
C6A	0.0155 (7)	0.0161 (7)	0.0175 (7)	0.0012 (5)	0.0065 (6)	−0.0002 (5)
C7A	0.0185 (7)	0.0174 (7)	0.0153 (6)	0.0021 (6)	0.0056 (6)	0.0005 (5)
C8A	0.0183 (7)	0.0208 (7)	0.0147 (6)	−0.0002 (6)	0.0047 (6)	−0.0002 (5)
C9A	0.0208 (8)	0.0220 (7)	0.0164 (7)	−0.0005 (6)	0.0058 (6)	0.0019 (6)
C10A	0.0166 (7)	0.0217 (7)	0.0202 (7)	−0.0015 (6)	0.0064 (6)	−0.0011 (6)
C11A	0.0162 (7)	0.0155 (7)	0.0185 (7)	0.0015 (5)	0.0061 (6)	−0.0007 (5)
C12A	0.0171 (7)	0.0157 (7)	0.0193 (7)	0.0023 (5)	0.0078 (6)	0.0000 (5)
C13A	0.0190 (7)	0.0160 (7)	0.0185 (7)	0.0042 (6)	0.0076 (6)	−0.0001 (5)
C14A	0.0172 (7)	0.0190 (7)	0.0236 (7)	0.0002 (6)	0.0088 (6)	−0.0010 (6)
C15A	0.0226 (8)	0.0208 (8)	0.0264 (8)	0.0029 (6)	0.0145 (7)	0.0024 (6)
C16A	0.0235 (8)	0.0252 (8)	0.0184 (7)	0.0038 (6)	0.0099 (6)	0.0014 (6)
C17A	0.0177 (7)	0.0184 (7)	0.0200 (7)	0.0027 (6)	0.0074 (6)	−0.0008 (5)
C18A	0.0253 (8)	0.0243 (8)	0.0257 (8)	0.0001 (6)	0.0158 (7)	0.0025 (6)
C19A	0.0198 (8)	0.0420 (10)	0.0378 (9)	−0.0093 (7)	0.0152 (7)	−0.0138 (8)
C20A	0.0226 (8)	0.0380 (9)	0.0156 (7)	−0.0009 (7)	0.0064 (6)	0.0028 (6)
C21A	0.0188 (8)	0.0230 (8)	0.0218 (7)	−0.0007 (6)	0.0052 (6)	−0.0039 (6)
C22A	0.0235 (8)	0.0286 (8)	0.0330 (8)	−0.0006 (7)	0.0162 (7)	0.0025 (7)
C23A	0.0224 (8)	0.0403 (10)	0.0166 (7)	−0.0001 (7)	0.0072 (6)	0.0002 (6)
O1B	0.0171 (5)	0.0371 (6)	0.0154 (5)	0.0035 (5)	0.0053 (4)	−0.0032 (4)
O2B	0.0181 (5)	0.0317 (6)	0.0179 (5)	0.0030 (4)	0.0085 (4)	−0.0004 (4)
O3B	0.0161 (5)	0.0345 (7)	0.0276 (6)	0.0042 (5)	0.0036 (5)	0.0039 (5)
O4B	0.0188 (5)	0.0312 (6)	0.0133 (5)	0.0019 (4)	0.0044 (4)	−0.0025 (4)
O5B	0.0158 (5)	0.0258 (6)	0.0169 (5)	0.0038 (4)	0.0042 (4)	0.0001 (4)
O6B	0.0224 (6)	0.0326 (6)	0.0266 (6)	0.0046 (5)	0.0144 (5)	−0.0028 (5)
O7B	0.0169 (5)	0.0304 (6)	0.0142 (5)	0.0040 (4)	0.0059 (4)	0.0000 (4)
C1B	0.0180 (7)	0.0176 (7)	0.0190 (7)	−0.0025 (6)	0.0081 (6)	0.0008 (5)
C2B	0.0165 (7)	0.0186 (7)	0.0267 (8)	−0.0001 (6)	0.0101 (6)	0.0022 (6)
C3B	0.0137 (7)	0.0190 (7)	0.0259 (8)	−0.0006 (6)	0.0046 (6)	0.0042 (6)
C4B	0.0190 (8)	0.0229 (8)	0.0172 (7)	−0.0018 (6)	0.0026 (6)	0.0009 (6)
C5B	0.0171 (7)	0.0170 (7)	0.0191 (7)	−0.0017 (6)	0.0070 (6)	−0.0001 (5)
C6B	0.0153 (7)	0.0158 (7)	0.0179 (7)	−0.0023 (5)	0.0057 (6)	0.0007 (5)
C7B	0.0155 (7)	0.0194 (7)	0.0141 (6)	−0.0038 (5)	0.0054 (5)	0.0006 (5)
C8B	0.0176 (7)	0.0185 (7)	0.0138 (6)	−0.0021 (6)	0.0054 (6)	−0.0005 (5)
C9B	0.0170 (7)	0.0174 (7)	0.0151 (6)	−0.0026 (5)	0.0079 (6)	0.0008 (5)
C10B	0.0144 (7)	0.0186 (7)	0.0170 (6)	0.0006 (5)	0.0055 (6)	0.0015 (5)
C11B	0.0150 (7)	0.0147 (7)	0.0183 (7)	0.0000 (5)	0.0070 (6)	0.0015 (5)
C12B	0.0178 (7)	0.0145 (7)	0.0172 (6)	−0.0008 (5)	0.0089 (6)	−0.0001 (5)
C13B	0.0180 (7)	0.0143 (7)	0.0167 (6)	−0.0014 (5)	0.0066 (6)	−0.0005 (5)
C14B	0.0151 (7)	0.0168 (7)	0.0245 (7)	0.0006 (5)	0.0089 (6)	−0.0006 (6)
C15B	0.0218 (8)	0.0167 (7)	0.0258 (7)	−0.0012 (6)	0.0159 (6)	−0.0017 (6)
C16B	0.0212 (8)	0.0206 (7)	0.0175 (7)	−0.0001 (6)	0.0098 (6)	−0.0006 (5)
C17B	0.0169 (7)	0.0166 (7)	0.0173 (6)	−0.0006 (5)	0.0076 (6)	0.0006 (5)
C18B	0.0238 (8)	0.0286 (8)	0.0273 (8)	0.0000 (6)	0.0164 (7)	−0.0014 (6)
C19B	0.0163 (8)	0.0273 (9)	0.0360 (9)	0.0032 (6)	0.0084 (7)	0.0064 (7)
C20B	0.0307 (9)	0.0356 (9)	0.0151 (7)	0.0042 (7)	0.0053 (7)	−0.0030 (6)
C21B	0.0168 (7)	0.0251 (8)	0.0208 (7)	0.0016 (6)	0.0045 (6)	0.0028 (6)
C22B	0.0232 (9)	0.0388 (10)	0.0357 (9)	0.0053 (7)	0.0165 (7)	−0.0035 (7)
C23B	0.0224 (8)	0.0412 (10)	0.0152 (7)	0.0045 (7)	0.0063 (6)	−0.0010 (6)

### Geometric parameters (Å, °)

**Table:**

O1A—C9A	1.2341 (17)	O1B—C9B	1.2404 (16)
O2A—C1A	1.3631 (17)	O2B—C1B	1.3643 (17)
O2A—C18A	1.4324 (17)	O2B—C18B	1.4282 (18)
O3A—C3A	1.3668 (17)	O3B—C3B	1.3672 (18)
O3A—C19A	1.430 (2)	O3B—C19B	1.433 (2)
O4A—C5A	1.3600 (17)	O4B—C5B	1.3631 (18)
O4A—C20A	1.4327 (16)	O4B—C20B	1.4324 (17)
O5A—C13A	1.3607 (16)	O5B—C13B	1.3607 (16)
O5A—C21A	1.4314 (17)	O5B—C21B	1.4379 (17)
O6A—C15A	1.3661 (18)	O6B—C15B	1.3716 (17)
O6A—C22A	1.4277 (19)	O6B—C22B	1.4316 (19)
O7A—C17A	1.3637 (17)	O7B—C17B	1.3648 (17)
O7A—C23A	1.4294 (17)	O7B—C23B	1.4357 (17)
C1A—C2A	1.391 (2)	C1B—C2B	1.396 (2)
C1A—C6A	1.418 (2)	C1B—C6B	1.410 (2)
C2A—C3A	1.391 (2)	C2B—C3B	1.388 (2)
C2A—H2AA	0.9300	C2B—H2BA	0.9300
C3A—C4A	1.393 (2)	C3B—C4B	1.390 (2)
C4A—C5A	1.383 (2)	C4B—C5B	1.384 (2)
C4A—H4AA	0.9300	C4B—H4BA	0.9300
C5A—C6A	1.4250 (19)	C5B—C6B	1.4194 (19)
C6A—C7A	1.451 (2)	C6B—C7B	1.4518 (19)
C7A—C8A	1.340 (2)	C7B—C8B	1.346 (2)
C7A—H7AA	0.9300	C7B—H7BA	0.9300
C8A—C9A	1.475 (2)	C8B—C9B	1.4717 (19)
C8A—H8AA	0.9300	C8B—H8BA	0.9300
C9A—C10A	1.465 (2)	C9B—C10B	1.474 (2)
C10A—C11A	1.342 (2)	C10B—C11B	1.3484 (19)
C10A—H10A	0.9300	C10B—H10B	0.9300
C11A—C12A	1.449 (2)	C11B—C12B	1.4514 (19)
C11A—H11A	0.9300	C11B—H11B	0.9300
C12A—C13A	1.408 (2)	C12B—C13B	1.413 (2)
C12A—C17A	1.4186 (19)	C12B—C17B	1.4238 (19)
C13A—C14A	1.396 (2)	C13B—C14B	1.397 (2)
C14A—C15A	1.390 (2)	C14B—C15B	1.389 (2)
C14A—H14A	0.9300	C14B—H14B	0.9300
C15A—C16A	1.392 (2)	C15B—C16B	1.391 (2)
C16A—C17A	1.382 (2)	C16B—C17B	1.382 (2)
C16A—H16A	0.9300	C16B—H16B	0.9300
C18A—H18A	0.9600	C18B—H18D	0.9600
C18A—H18B	0.9600	C18B—H18E	0.9600
C18A—H18C	0.9600	C18B—H18F	0.9600
C19A—H19A	0.9600	C19B—H19D	0.9600
C19A—H19B	0.9600	C19B—H19E	0.9600
C19A—H19C	0.9600	C19B—H19F	0.9600
C20A—H20A	0.9600	C20B—H20D	0.9600
C20A—H20B	0.9600	C20B—H20E	0.9600
C20A—H20C	0.9600	C20B—H20F	0.9600
C21A—H21A	0.9600	C21B—H21D	0.9600
C21A—H21B	0.9600	C21B—H21E	0.9600
C21A—H21C	0.9600	C21B—H21G	0.9600
C22A—H22A	0.9600	C22B—H22D	0.9600
C22A—H22B	0.9600	C22B—H22G	0.9600
C22A—H22C	0.9600	C22B—H22E	0.9600
C23A—H23A	0.9600	C23B—H23G	0.9600
C23A—H23B	0.9600	C23B—H23D	0.9600
C23A—H23C	0.9600	C23B—H23E	0.9600
			
C1A—O2A—C18A	118.11 (11)	C1B—O2B—C18B	118.46 (12)
C3A—O3A—C19A	117.06 (12)	C3B—O3B—C19B	117.61 (12)
C5A—O4A—C20A	118.13 (11)	C5B—O4B—C20B	117.96 (12)
C13A—O5A—C21A	118.64 (11)	C13B—O5B—C21B	118.29 (11)
C15A—O6A—C22A	118.28 (12)	C15B—O6B—C22B	117.81 (12)
C17A—O7A—C23A	118.30 (12)	C17B—O7B—C23B	117.80 (11)
O2A—C1A—C2A	122.34 (13)	O2B—C1B—C2B	122.27 (13)
O2A—C1A—C6A	115.35 (12)	O2B—C1B—C6B	115.09 (12)
C2A—C1A—C6A	122.31 (13)	C2B—C1B—C6B	122.64 (13)
C1A—C2A—C3A	118.70 (13)	C3B—C2B—C1B	118.43 (14)
C1A—C2A—H2AA	120.6	C3B—C2B—H2BA	120.8
C3A—C2A—H2AA	120.6	C1B—C2B—H2BA	120.8
O3A—C3A—C2A	123.44 (13)	O3B—C3B—C2B	123.77 (14)
O3A—C3A—C4A	115.15 (13)	O3B—C3B—C4B	114.82 (13)
C2A—C3A—C4A	121.41 (13)	C2B—C3B—C4B	121.41 (13)
C5A—C4A—C3A	119.37 (13)	C5B—C4B—C3B	119.32 (13)
C5A—C4A—H4AA	120.3	C5B—C4B—H4BA	120.3
C3A—C4A—H4AA	120.3	C3B—C4B—H4BA	120.3
O4A—C5A—C4A	123.06 (13)	O4B—C5B—C4B	122.72 (13)
O4A—C5A—C6A	115.16 (12)	O4B—C5B—C6B	115.21 (12)
C4A—C5A—C6A	121.78 (13)	C4B—C5B—C6B	122.04 (13)
C1A—C6A—C5A	116.41 (13)	C1B—C6B—C5B	116.16 (13)
C1A—C6A—C7A	118.89 (12)	C1B—C6B—C7B	119.47 (13)
C5A—C6A—C7A	124.64 (13)	C5B—C6B—C7B	124.37 (13)
C8A—C7A—C6A	130.38 (13)	C8B—C7B—C6B	129.17 (13)
C8A—C7A—H7AA	114.8	C8B—C7B—H7BA	115.4
C6A—C7A—H7AA	114.8	C6B—C7B—H7BA	115.4
C7A—C8A—C9A	120.69 (13)	C7B—C8B—C9B	121.91 (13)
C7A—C8A—H8AA	119.7	C7B—C8B—H8BA	119.0
C9A—C8A—H8AA	119.7	C9B—C8B—H8BA	119.0
O1A—C9A—C10A	119.07 (13)	O1B—C9B—C8B	121.64 (13)
O1A—C9A—C8A	120.49 (14)	O1B—C9B—C10B	118.57 (12)
C10A—C9A—C8A	120.42 (12)	C8B—C9B—C10B	119.77 (12)
C11A—C10A—C9A	123.14 (13)	C11B—C10B—C9B	123.21 (13)
C11A—C10A—H10A	118.4	C11B—C10B—H10B	118.4
C9A—C10A—H10A	118.4	C9B—C10B—H10B	118.4
C10A—C11A—C12A	131.90 (14)	C10B—C11B—C12B	131.73 (13)
C10A—C11A—H11A	114.0	C10B—C11B—H11B	114.1
C12A—C11A—H11A	114.0	C12B—C11B—H11B	114.1
C13A—C12A—C17A	116.96 (13)	C13B—C12B—C17B	116.47 (12)
C13A—C12A—C11A	125.77 (13)	C13B—C12B—C11B	125.83 (12)
C17A—C12A—C11A	117.27 (13)	C17B—C12B—C11B	117.65 (12)
O5A—C13A—C14A	122.67 (13)	O5B—C13B—C14B	122.59 (13)
O5A—C13A—C12A	115.42 (13)	O5B—C13B—C12B	115.46 (12)
C14A—C13A—C12A	121.91 (13)	C14B—C13B—C12B	121.95 (13)
C15A—C14A—C13A	118.66 (14)	C15B—C14B—C13B	118.61 (13)
C15A—C14A—H14A	120.7	C15B—C14B—H14B	120.7
C13A—C14A—H14A	120.7	C13B—C14B—H14B	120.7
O6A—C15A—C14A	123.94 (14)	O6B—C15B—C14B	123.52 (13)
O6A—C15A—C16A	114.52 (13)	O6B—C15B—C16B	114.49 (13)
C14A—C15A—C16A	121.54 (14)	C14B—C15B—C16B	121.99 (13)
C17A—C16A—C15A	119.10 (14)	C17B—C16B—C15B	118.61 (13)
C17A—C16A—H16A	120.5	C17B—C16B—H16B	120.7
C15A—C16A—H16A	120.5	C15B—C16B—H16B	120.7
O7A—C17A—C16A	123.15 (13)	O7B—C17B—C16B	122.87 (12)
O7A—C17A—C12A	115.01 (13)	O7B—C17B—C12B	114.76 (12)
C16A—C17A—C12A	121.83 (14)	C16B—C17B—C12B	122.37 (13)
O2A—C18A—H18A	109.5	O2B—C18B—H18D	109.5
O2A—C18A—H18B	109.5	O2B—C18B—H18E	109.5
H18A—C18A—H18B	109.5	H18D—C18B—H18E	109.5
O2A—C18A—H18C	109.5	O2B—C18B—H18F	109.5
H18A—C18A—H18C	109.5	H18D—C18B—H18F	109.5
H18B—C18A—H18C	109.5	H18E—C18B—H18F	109.5
O3A—C19A—H19A	109.5	O3B—C19B—H19D	109.5
O3A—C19A—H19B	109.5	O3B—C19B—H19E	109.5
H19A—C19A—H19B	109.5	H19D—C19B—H19E	109.5
O3A—C19A—H19C	109.5	O3B—C19B—H19F	109.5
H19A—C19A—H19C	109.5	H19D—C19B—H19F	109.5
H19B—C19A—H19C	109.5	H19E—C19B—H19F	109.5
O4A—C20A—H20A	109.5	O4B—C20B—H20D	109.5
O4A—C20A—H20B	109.5	O4B—C20B—H20E	109.5
H20A—C20A—H20B	109.5	H20D—C20B—H20E	109.5
O4A—C20A—H20C	109.5	O4B—C20B—H20F	109.5
H20A—C20A—H20C	109.5	H20D—C20B—H20F	109.5
H20B—C20A—H20C	109.5	H20E—C20B—H20F	109.5
O5A—C21A—H21A	109.5	O5B—C21B—H21D	109.5
O5A—C21A—H21B	109.5	O5B—C21B—H21E	109.5
H21A—C21A—H21B	109.5	H21D—C21B—H21E	109.5
O5A—C21A—H21C	109.5	O5B—C21B—H21G	109.5
H21A—C21A—H21C	109.5	H21D—C21B—H21G	109.5
H21B—C21A—H21C	109.5	H21E—C21B—H21G	109.5
O6A—C22A—H22A	109.5	O6B—C22B—H22D	109.5
O6A—C22A—H22B	109.5	O6B—C22B—H22G	109.5
H22A—C22A—H22B	109.5	H22D—C22B—H22G	109.5
O6A—C22A—H22C	109.5	O6B—C22B—H22E	109.5
H22A—C22A—H22C	109.5	H22D—C22B—H22E	109.5
H22B—C22A—H22C	109.5	H22G—C22B—H22E	109.5
O7A—C23A—H23A	109.5	O7B—C23B—H23G	109.5
O7A—C23A—H23B	109.5	O7B—C23B—H23D	109.5
H23A—C23A—H23B	109.5	H23G—C23B—H23D	109.5
O7A—C23A—H23C	109.5	O7B—C23B—H23E	109.5
H23A—C23A—H23C	109.5	H23G—C23B—H23E	109.5
H23B—C23A—H23C	109.5	H23D—C23B—H23E	109.5
			
C18A—O2A—C1A—C2A	−1.5 (2)	C18B—O2B—C1B—C2B	−0.7 (2)
C18A—O2A—C1A—C6A	178.45 (12)	C18B—O2B—C1B—C6B	179.23 (13)
O2A—C1A—C2A—C3A	−179.22 (13)	O2B—C1B—C2B—C3B	178.78 (13)
C6A—C1A—C2A—C3A	0.8 (2)	C6B—C1B—C2B—C3B	−1.1 (2)
C19A—O3A—C3A—C2A	−7.2 (2)	C19B—O3B—C3B—C2B	−5.5 (2)
C19A—O3A—C3A—C4A	173.26 (13)	C19B—O3B—C3B—C4B	174.68 (13)
C1A—C2A—C3A—O3A	179.02 (13)	C1B—C2B—C3B—O3B	−179.09 (14)
C1A—C2A—C3A—C4A	−1.4 (2)	C1B—C2B—C3B—C4B	0.7 (2)
O3A—C3A—C4A—C5A	−179.30 (13)	O3B—C3B—C4B—C5B	−179.89 (13)
C2A—C3A—C4A—C5A	1.1 (2)	C2B—C3B—C4B—C5B	0.3 (2)
C20A—O4A—C5A—C4A	4.1 (2)	C20B—O4B—C5B—C4B	−0.6 (2)
C20A—O4A—C5A—C6A	−176.28 (13)	C20B—O4B—C5B—C6B	177.44 (13)
C3A—C4A—C5A—O4A	179.50 (13)	C3B—C4B—C5B—O4B	176.94 (13)
C3A—C4A—C5A—C6A	−0.1 (2)	C3B—C4B—C5B—C6B	−0.9 (2)
O2A—C1A—C6A—C5A	−179.87 (12)	O2B—C1B—C6B—C5B	−179.37 (12)
C2A—C1A—C6A—C5A	0.1 (2)	C2B—C1B—C6B—C5B	0.5 (2)
O2A—C1A—C6A—C7A	2.78 (19)	O2B—C1B—C6B—C7B	1.53 (19)
C2A—C1A—C6A—C7A	−177.23 (13)	C2B—C1B—C6B—C7B	−178.56 (13)
O4A—C5A—C6A—C1A	179.88 (12)	O4B—C5B—C6B—C1B	−177.51 (12)
C4A—C5A—C6A—C1A	−0.5 (2)	C4B—C5B—C6B—C1B	0.5 (2)
O4A—C5A—C6A—C7A	−2.9 (2)	O4B—C5B—C6B—C7B	1.5 (2)
C4A—C5A—C6A—C7A	176.72 (14)	C4B—C5B—C6B—C7B	179.55 (14)
C1A—C6A—C7A—C8A	−179.23 (15)	C1B—C6B—C7B—C8B	−169.16 (14)
C5A—C6A—C7A—C8A	3.7 (2)	C5B—C6B—C7B—C8B	11.8 (2)
C6A—C7A—C8A—C9A	−175.78 (14)	C6B—C7B—C8B—C9B	179.71 (13)
C7A—C8A—C9A—O1A	1.1 (2)	C7B—C8B—C9B—O1B	0.5 (2)
C7A—C8A—C9A—C10A	179.37 (14)	C7B—C8B—C9B—C10B	179.49 (13)
O1A—C9A—C10A—C11A	176.83 (15)	O1B—C9B—C10B—C11B	169.13 (14)
C8A—C9A—C10A—C11A	−1.4 (2)	C8B—C9B—C10B—C11B	−9.9 (2)
C9A—C10A—C11A—C12A	−178.22 (15)	C9B—C10B—C11B—C12B	−173.57 (14)
C10A—C11A—C12A—C13A	−6.6 (3)	C10B—C11B—C12B—C13B	−0.5 (3)
C10A—C11A—C12A—C17A	173.70 (15)	C10B—C11B—C12B—C17B	176.71 (15)
C21A—O5A—C13A—C14A	−0.1 (2)	C21B—O5B—C13B—C14B	−0.9 (2)
C21A—O5A—C13A—C12A	−179.80 (12)	C21B—O5B—C13B—C12B	179.03 (12)
C17A—C12A—C13A—O5A	179.72 (12)	C17B—C12B—C13B—O5B	179.84 (12)
C11A—C12A—C13A—O5A	0.1 (2)	C11B—C12B—C13B—O5B	−2.9 (2)
C17A—C12A—C13A—C14A	0.0 (2)	C17B—C12B—C13B—C14B	−0.3 (2)
C11A—C12A—C13A—C14A	−179.66 (14)	C11B—C12B—C13B—C14B	177.01 (13)
O5A—C13A—C14A—C15A	−179.28 (13)	O5B—C13B—C14B—C15B	179.29 (13)
C12A—C13A—C14A—C15A	0.4 (2)	C12B—C13B—C14B—C15B	−0.6 (2)
C22A—O6A—C15A—C14A	−0.7 (2)	C22B—O6B—C15B—C14B	1.6 (2)
C22A—O6A—C15A—C16A	179.22 (13)	C22B—O6B—C15B—C16B	−178.63 (14)
C13A—C14A—C15A—O6A	179.70 (14)	C13B—C14B—C15B—O6B	−179.60 (13)
C13A—C14A—C15A—C16A	−0.2 (2)	C13B—C14B—C15B—C16B	0.7 (2)
O6A—C15A—C16A—C17A	179.69 (13)	O6B—C15B—C16B—C17B	−179.62 (13)
C14A—C15A—C16A—C17A	−0.4 (2)	C14B—C15B—C16B—C17B	0.1 (2)
C23A—O7A—C17A—C16A	0.8 (2)	C23B—O7B—C17B—C16B	−4.9 (2)
C23A—O7A—C17A—C12A	−178.34 (13)	C23B—O7B—C17B—C12B	175.67 (13)
C15A—C16A—C17A—O7A	−178.23 (14)	C15B—C16B—C17B—O7B	179.56 (13)
C15A—C16A—C17A—C12A	0.8 (2)	C15B—C16B—C17B—C12B	−1.0 (2)
C13A—C12A—C17A—O7A	178.48 (12)	C13B—C12B—C17B—O7B	−179.45 (12)
C11A—C12A—C17A—O7A	−1.82 (19)	C11B—C12B—C17B—O7B	3.04 (18)
C13A—C12A—C17A—C16A	−0.6 (2)	C13B—C12B—C17B—C16B	1.1 (2)
C11A—C12A—C17A—C16A	179.06 (13)	C11B—C12B—C17B—C16B	−176.40 (13)

### Hydrogen-bond geometry (Å, °)

**Table:**

Cg1, Cg2 and Cg3 are the centroids of the C1A–C6A, C12A–C17A and C12B–C17B rings, respectively

**Table:**

*D*—H···*A*	*D*—H	H···*A*	*D*···*A*	*D*—H···*A*
C7A—H7AA···O1A	0.93	2.42	2.787 (2)	104
C7A—H7AA···O2A	0.93	2.29	2.704 (2)	106
C8A—H8AA···O4A	0.93	2.18	2.7844 (17)	121
C7B—H7BA···O1B	0.93	2.49	2.8351 (19)	102
C7B—H7BA···O2B	0.93	2.33	2.704 (2)	104
C8B—H8BA···O4B	0.93	2.16	2.7643 (18)	121
C10A—H10A···O5A	0.93	2.26	2.852 (2)	121
C10B—H10B···O5B	0.93	2.26	2.855 (2)	121
C11A—H11A···O7A	0.93	2.23	2.6599 (17)	108
C11B—H11B···O7B	0.93	2.23	2.6700 (17)	108
C20A—H20C···O1B	0.96	2.47	3.339 (2)	151
C20B—H20E···O1A^i^	0.96	2.37	3.0238 (19)	125
C23A—H23C···O1A^ii^	0.96	2.39	3.319 (2)	162
C23B—H23D···O1B^ii^	0.96	2.41	3.262 (2)	148
C18A—H18C···Cg1^iii^	0.96	2.65	3.4503 (17)	141
C21A—H21C···Cg2^iv^	0.96	2.94	3.5813 (18)	126
C21B—H21E···Cg3^v^	0.96	2.72	3.5809 (16)	150

Symmetry codes:  (i) *x*+1, *y*, *z*+1; (ii) *x*, −*y*+1/2, *z*+1/2; (iii) −*x*+1, −*y*+1, −*z*; (iv) −*x*, −*y*, −*z*; (v) −*x*−1, −*y*+1, −*z*−1.
